# Synthesis of functionalized macrocyclic derivatives of trioxabicyclo[3.3.0]nonadiene

**DOI:** 10.3762/bjoc.8.83

**Published:** 2012-05-15

**Authors:** Sabine Leber, Gert Kollenz, Curt Wentrup

**Affiliations:** 1 Institute of Chemistry, Karl-Franzens University of Graz, Heinrichstrasse 28, A-8010 Graz, Austria; 2 School of Chemistry and Molecular Biosciences, The University of Queensland, Brisbane, QLD 4072, Australia

**Keywords:** bisdioxine, macrocyclic amines, macrocyclic nitro compounds, tetraoxaadamantane

## Abstract

C_72_-Macrocyclic systems functionalized with nitroaryl and arylamino groups were synthesized from the bisdioxine diacid dichloride 1,3,5,7-tetra-*tert*-butyl-2,6,9-trioxabicyclo[3.3.1]nona-3,7-diene-4,8-dicarbonyl dichloride (**3**).

## Introduction

**Scheme 1:**
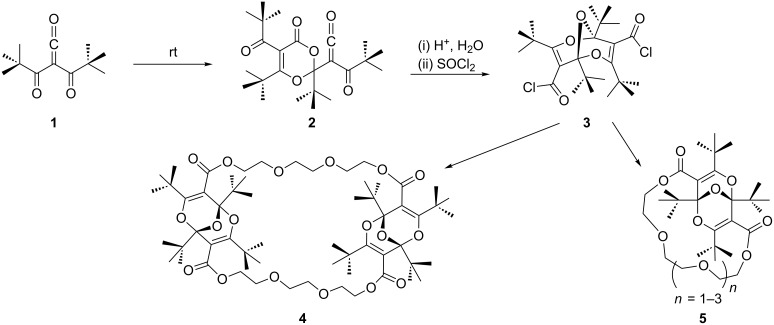
Synthesis of macrocyclic bisdioxine derivatives (*R,S*-form of **4** and *S*-form of **5** shown; see Supporting Information File 1 for details).

The concave, axially chiral [1], bridged bisdioxine diacid dichloride **3** is obtained by acid hydrolysis and subsequent chlorination of the surprisingly stable α-oxoketene **2**, itself obtained by dimerization of dipivaloylketene (**1**) (Scheme 1) [2–3]. The concave structure of **3** and its derivatives together with the sterically hindering *tert*-butyl groups make it an interesting spacer group, and it thus has been applied successfully in syntheses of several macrocyclic polyether and polymethyleneoxy rings containing one, two or three bisdioxine units, e.g., **4** and **5** (Scheme 1) [4–5]. Capping of a calix[6]arene with the bisdioxine unit has also been achieved recently [6]. Some of these materials exhibit pronounced complexation of metal ions, such as Cs^+^, Hg^2+^, Cu^2+^, Ag^+^, and Au^3+^ [5–7].

It may also be possible to stabilize reactive intermediates and unusual functional groups in the concave interiors of the bisdioxine-derived macrocycles. Okazaki and co-workers designed bowl-shaped [8] and lantern-shaped [9] molecules containing a functionalized aryl group, which allowed the preparation of, among other things, stable simple enols [8] and a variety of unusual sulfur [9], selenium [10–11] and germanium [12] species. Clearly, the bisdioxine macrocycles such as **4** and **5** will not be nearly as rigid, but they may nevertheless exert some steric protection. Herein we report the realisation of the first step toward this end, the preparation of functionalized macrocyclic bisdioxine derivatives.

## Results and Discussion

**Scheme 2:**
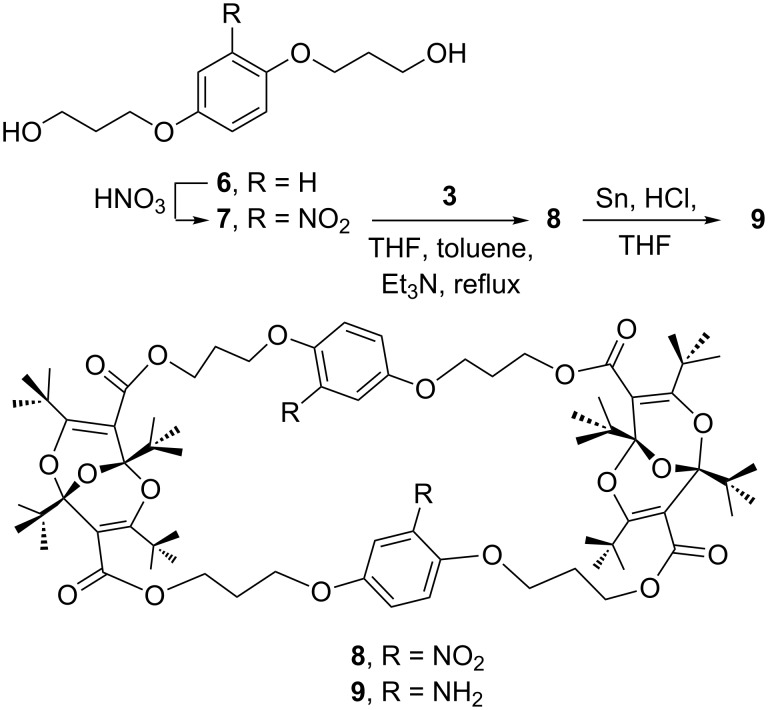
Synthesis of the 1,2,4-trisubstituted aryl derivatives.

**Scheme 3:**
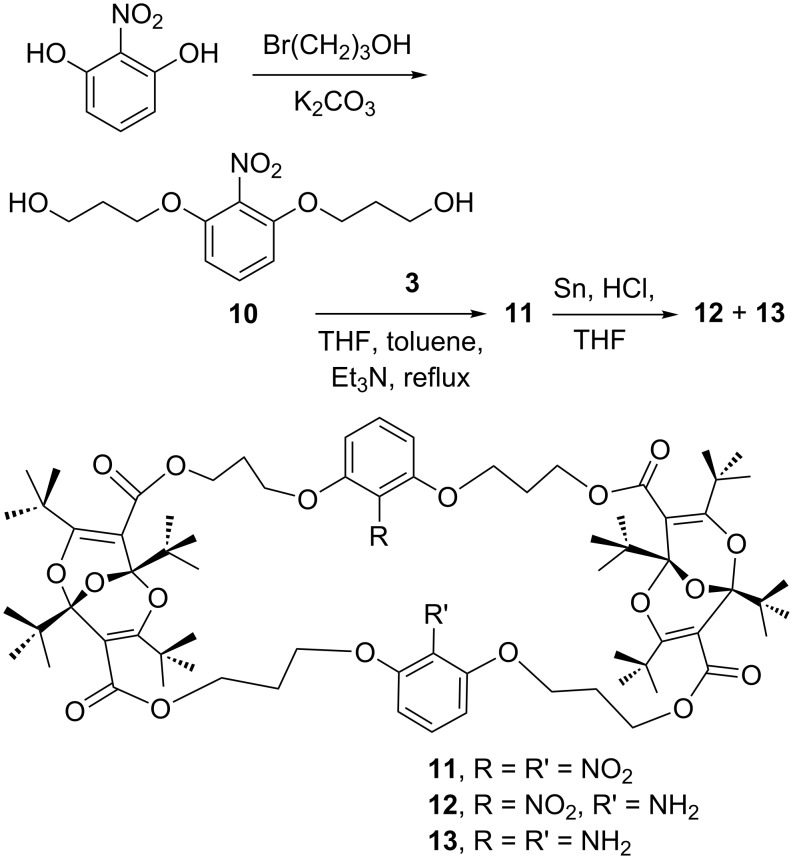
Synthesis of the 1,2,3-trisubstituted aryl derivatives.

Functional group manipulation on aromatic rings often starts with the nitro group. Therefore, a synthesis of suitable nitro-aromatic diols for combination with the diacid dichloride **3** was required. The desired 2-nitro-1,4-phenylene derivative **7** was prepared by treatment of hydroquinone with 3-bromopropanol followed by nitration of the resulting diol **6** (Scheme 2). The isomeric 1,2,3-trisubstituted aromatic **10** was obtained by etherification of nitroresorcinol (Scheme 3).

The two diols **7** and **10** reacted readily with the diacid dichloride **3** in boiling toluene in the presence of triethylamine to afford the 2:2 adducts **8** and **11**, respectively (Scheme 2 and Scheme 3). These compounds were characterized by elemental analysis and their ^1^H- and ^13^C NMR spectra. All proton and carbon resonances could be assigned by comparison with data for other, related, macrocycles [1–7] (see Supporting Information File 2 for spectral details). Since **3** is chiral (existing in enantiomeric *R* and *S* forms) [1,4], the nitro compounds **8** and **11** must also be chiral, i.e., they must exist as mixtures of diastereoisomeric forms (see the Supporting Information File 1 for drawings of the principal structures). This will also be the case for the derivatives described below. The splitting of several signals in the NMR spectra may be ascribed to the presence of mixtures of diastereoisomers and conformers.

### Reduction

Several methods were attempted for the reduction of the nitro groups in **8** and **11**. Reductions with NaBH_4_ and sulfur [13] or with ammonium formate and Pd/C under microwave irradiation [14] were unsuccessful. However, compound **8** was completely reduced to the diamine **9** by using the classical reduction with Sn and HCl (Scheme 2). The reaction was complete in 1 h. Under the same conditions it took 3 h for the 2,6-disubstituted nitro compound **11** to be completely consumed. Compound **11** is more compact than **8** (see below), and the reduction of **11** was evidently more difficult. Although the product was largely the desired diamine **13**, mass spectrometry indicated the presence of the singly reduced nitro derivative **12** as an impurity (Scheme 3).

**Scheme 4:**
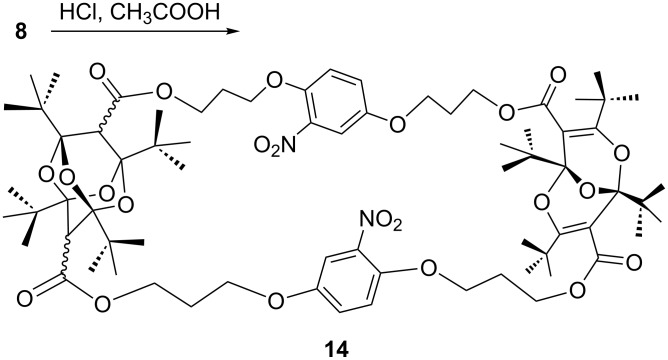
Synthesis of the tetraoxaadamantane derivative **14**.

A remarkable reaction is the ready conversion of macrocyclic as well as open-chain bisdioxine derivatives to 2,4,6,8-tetraoxaadamantanes on acid hydrolysis [4,7,15]. This transformation was also achieved with the dinitro compound **8**, which yielded the mono-tetraoxaadamantane derivative **14** (Scheme 4), but all attempts to convert the second bisdioxine unit were fruitless, presumably due to steric hindrance. Force-field calculations [16] indicate that the internal cavity is much smaller in **11** than in **8**, and in fact it was not possible to prepare a tetraoxaadamantane derivative of nitro compound **11**. There is a significant cavity in **8**, obviously large enough to form one tetraoxaadamantane derivative, but this reduces the available space, with the consequence that the attack by a water molecule on the second bisdioxine unit from the concave inside of the macrocycle does not take place.

## Conclusion

The difficulty of reduction of the nitro compounds, in particular the 1,2,3-trisubstituted compound **11**, as well as the conversion of only one of the bisdioxine units in **8** to a tetraoxaadamantane suggests that these macrocycles provide steric protection of the functional groups. The cavity in **8** is obviously large enough to permit the formation of one tetraoxaadamantane unit, but this will reduce the available space, with the consequence that the attack by a water molecule on the second bisdioxine unit from the concave inside of the macrocycle does not take place. Moreover, no tetraoxaadamantane derivative of nitro compound **11** was obtainable. Here, the cavity is too small for the formation of a tetraoxaadamantane. Further investigations of reactivity and functional group manipulation in the macrocycles described herein are foreseen.

## Experimental

**General**. All solvents were dried to achieve the minimum degree of water content. Melting points are uncorrected. Dry-column flash chromatography (DCFC) was performed according to a literature method [17] by using silicagel 6H from Merck, Darmstadt and eluting with CH_2_Cl_2_/MeOH 100:5, unless indicated otherwise. Thin-layer chromatography (TLC) was performed on silica gel. LC–MS was performed by using a mixture of 77% CH_3_CN, 18% H_2_O, and 5% MeOH as mobile phase, unless otherwise indicated, and an atmospheric-pressure chemical ionization source. All NMR spectra were recorded for CDCl_3_ solutions. Assignments of NMR signals for bisdioxine and tetraoxaadamantane units were made in agreement with previously reported data [1–7,15].

**1,3,5,7-Tetra-*tert*-butyl-2,6,9-trioxabicyclo[3.3.1]nona-3,7-diene-4,8-dicarbonyl dichloride (3):** Flash vacuum thermolysis of 5-*tert*-butyl-4-pivaloylfuran-2,3-dione generates dipivaloylketene (**1**), which slowly dimerizes to the dimeric oxoketene **2** at room temperature [2–3]. Hydrolysis and subsequent chlorination with thionyl chloride afforded the bisdioxine diacid dichloride **3** [3].

**4,4’-(2-Nitro-1,4-phenylene)bis(4-oxabutanol) (7):** Diol **6** was prepared according to the literature [18]. To a solution of **6** (750 mg, 3.32 mmol) in 65 mL of glacial acetic acid was added 22 mL of 37% HNO_3_ under stirring. The solution turned intensely yellow immediately. After being stirred for 30 min the mixture was diluted with 100 mL of water, neutralized with aq KOH, and extracted several times with CH_2_Cl_2_. The combined organic layers were dried over Na_2_SO_4_, filtered, and concentrated in vacuo. The resulting oily product (1.44 g) was purified by DCFC to yield 350 mg (39%) of intensely yellow crystals, mp 48–49 °C; ^1^H NMR δ 2.05–2.11 (m, 4H, CH_2_), 2.66 (s, br, 2H, OH), 3.86–3.93 (m, 4H, CH_2_OH), 4.12–4.15 (m, 2H, OCH_2_), 4.23–4.26 (m, 2H, OCH_2_), 7.05–7.15 (m, 2H, arom. H5, H6), 7.46–7.47 (m, 1H, arom. H3); ^13^C NMR δ 31.7 (CH_2_), 31.9 (CH_2_), 59.9 (CH_2_OH), 60.4 (CH_2_OH), 66.4 (OCH_2_), 68.4 (OCH_2_), 110.9 (arom. C3), 116.0 (arom. C6), 121.7 (arom. C5), 139.4 (arom. C2), 146.8 (arom. C1), 152.3 (arom. C4); the NMR spectra were assigned on the basis of comparison with standard data compilations; LC–MS (CH_2_Cl_2_) *m*/*z*: 271; Anal. calcd for C_12_H_17_NO_6_: C, 53.13; H, 6.32; N, 5.16; found: C, 53.30; H, 6.43; N, 5.10.

**Bis(2-nitro-1,4-phenylene)macrocycle 8:** A sample of diacid dichloride **3** (500 mg, 1.05 mmol) was dissolved in 25 mL of toluene. The diol **7** (285 mg, 1.05 mmol) was separately dissolved in 8 mL of toluene, and 1 mL of Et_3_N in 17 mL of THF was added. The two solutions were placed in separate dropping funnels attached to a flask containing 80 mL of toluene, fitted with a reflux condenser and protected from moisture. The apparatus was flushed with N_2_. The two solutions were simultaneously added dropwise to the toluene under reflux over a 3 h period, and the resulting mixture was heated under reflux for 20 h. After cooling to 60 °C and filtering on a folded filter, the resulting solution was evaporated, and the material so obtained was triturated with 5 mL diethyl ether to form a yellow precipitate. DCFC afforded 163 mg (23%) of yellow crystals, mp 264–266 °C dec; ^1^H NMR δ 1.04 (s, 36H, CH_3_(*t*-Bu)), 1.10–1.15 (36H, CH_3_(*t*-Bu)), 2.11–2.15 (m, 8H, CH_2_), 3.96–4.06 (m, 12H, CH_2_-O), 4.43–4.48 (m, 4H, CH_2_O), 6.90 (m, 2H, arom. H6), 7.02 (m, 2H, arom. H5), 7.34 (m, 2H, arom. H3); ^13^C NMR δ 24.6 (CH_3_), 28.0 (two signals, CH_2_), 28.6 (CH_3_), 37.3 (C(*t*-Bu)), 39.5 (C(*t*-Bu)), 61.0 and 61.3 (two signals, CH_2_O), 64.9 (two signals, CH_2_O), 66.3 (CH_2_O), 98.1 (bisdioxine C1/C5), 102.3 (two signals, bisdioxine C4/C8), 110.4 (arom. C3), 116.0 (arom. C6), 121.3 (arom. C5), 139.5 (arom. C2), 146.6 (arom. C1/C3), 152.1 (arom. C4/C6), 163.1 (three signals, bisdioxine C3/C7), 169.4 (three signals, CO); NMR spectra were assigned on the basis of previously reported data for related bisdioxine derivatives [3–7]; IR (KBr): 3000–2800, 1720, 1619, 1535 cm^−1^; LC–MS *m*/*z*: 1347.8 [M + H]^+^; Anal. calcd for C_72_H_102_N_2_O_22_: C, 64.16; H, 7.63; N, 2.08; found: C, 63.77; H, 7.68; N, 1.95.

**Bis(2-amino-1,4-phenylene)macrocycle 9:** A mixture of 100 mg (0.07 mmol) of **8** and 40 mg (2.4 mmol) of tin granules in 10 mL of THF was heated to reflux. Subsequently, 300 µL of conc. HCl was added dropwise, which resulted in strong gas evolution. The reaction was followed by TLC (CH_2_Cl_2_/MeOH 100:1), which indicated completion after 1 h. The reaction mixture was cooled, neutralized with 2 M NaOH, and filtered. The resulting solution was extracted with CH_2_Cl_2_ and concentrated, and the residue was triturated with diethyl ether, which caused the formation of a white precipitate. The product was purified by DCFC, eluting with hexane/diethyl ether/MeOH 100:30:2 to yield 10 mg (10%) of a slightly yellow solid. ^1^H NMR δ 1.04 (s, 36H, CH_3_(*t*-Bu)), 1.14 (s, 36H, CH_3_(*t*-Bu)), 2.06–2.08 (m, 8H, CH_2_), 3.84–4.01 (m, 12H, CH_2_O), 4.45–4.48 (m, 4H, CH_2_O), 6.10 (m, 2H, arom. H3), 6.26 (m, 2H, arom. H5), 6.52 (m, 2H, arom. H6); ^13^C NMR δ 24.6 (CH_3_(*t*-Bu)), 28.2 (CH_2_), 28.7 (CH_3_(*t-*Bu)), 37.3 (C(*t*-Bu)), 39.5 (C(*t*-Bu)), 61.6 (CH_2_O), 64.5 (CH_2_O), 68.2 (CH_2_O), 98.1 (bisdioxine C1/C5), 102.3 (bisdioxine C4/C8), 112.2 (arom.), 114.3 (arom.), 126.6 (arom.), 128.8 (arom.), 130.9 (arom.), 153.6 (arom. C4), 163.0 (bisdioxine C3/C7), 169.6 (CO); LC–MS *m*/*z*: 1287.8 [M + H]^+^; Anal. calcd for C_72_H_106_N_2_O_18_: C, 67.15; H, 8.30; N, 2.18; found: C, 67.42; H, 8.34; N, 2.16.

**4,4’-(2-Nitro-1,3-phenylene) bis(4-oxabutanol) (10):** A mixture of 2-nitroresorcinol (2.5 g, 16.1 mmol), 4.75 g (34.2 mmol) of 3-bromopropanol and 8 g (57.9 mmol) K_2_CO_3_ in 25 mL acetone was heated under reflux for 12 h under N_2_ with the exclusion of moisture. After cooling to rt, 100 mL of water was added, and the mixture was extracted with CH_2_Cl_2_. The organic phase was dried over Na_2_SO_4_ and concentrated in vacuo, and the resulting oily product was purified by DCFC, eluting with CH_2_Cl_2_/MeOH 20:1 to afford 1.18 g (27%) of light-yellow crystals, mp 72–73 °C; ^1^H NMR δ 1.99–2.05 (m, 4H, CH_2_), 2.18 (s, br, 2H, OH), 3.80–3.83 (m, 4H, C*H*_2_-OH), 4.20–4.23 (m, 4H, O-CH_2_), 6.64–6.66 (m, 2H, arom. H4/H6), 7.29–7.35 (m, 1H, arom. H5); ^13^C NMR δ 31.5 (CH_2_), 59.4 (CH_2_OH), 66.6 (O-CH_2_), 105.5 (arom. C4/C6), 131.3 (arom. C2), 132.3 (arom. C5), 151.3 (arom. C1/C3); LC–MS (CH_2_Cl_2_) *m*/*z*: 271; Anal. calcd for C_12_H_17_NO_6_: C, 53.13; H, 6.32; N, 5.16; found: C, 53.37; H, 6.42; N, 5.04.

**Bis(2-nitro-1,3-phenylene)macrocycle 11:** This compound was prepared from the diacid dichloride **3** and the diol **10** using the method described for **8**. Yield 176 mg (25%), white crystals, mp 300–302 °C dec; ^1^H NMR δ 1.03 (s, 36H, CH_3_(*t*-Bu)), 1.09 (s, 36H, CH_3_(*t*-Bu)), 2.11–2.13 (m, 8H, CH_2_), 4.00–4.06 (m, 12H, CH_2_O), 4.37–4.40 (m, 4H, CH_2_O), 6.44 (m, 4H, arom. H4), 7.24 (m, 2H, arom. H5); ^13^C NMR δ 24.5 (CH_3_-(*t*-Bu)), 27.9 (CH_2_), 28.4 (CH_3_(*t*-Bu)), 37.2 (C(*t*-Bu)), 39.4 (C(*t*-Bu)), 60.6 (CH_2_O), 65.4 and 65.4 (two signals, CH_2_O), 97.95 and 97.98 (two signals, bisdioxine C1/C5), 102.12, 102.14 (two signals, bisdioxine C4/C8), 105.1 (arom. C4), 131.1 (arom. C2), 132.2 (arom. C5), 150.8 (arom. C1), 162.8 (two signals, bisdioxine C3/C7), 169.2 (two signals, CO); IR (KBr) 2800–3000, 1721, 1615, 1541 cm^−1^; LC–MS *m*/*z*: 1347.5 [M + H]^+^; Anal. calcd for C_72_H_102_N_2_O_22_: C, 64.16; H, 7.63; N, 2.08; found: C, 64.32; H, 7.82; N, 2.07.

**Bis(2-amino-1,3-phenylene)macrocycle 13:** The dinitro compound **11** (44 mg; 0.03 mmol) was reduced with 20 mg (1.69 mmol) of tin granules in 5 mL of THF and 150 µL of conc. HCl, as described for the reduction of **8** above. It took 3 h for the starting material to be fully consumed, yielding 10 mg of the diamine as a white precipitate. ^1^H NMR δ 1.04–1.16 (m, 72H, *t*-Bu), 2.18 (m, 8H, CH_2_), 3.99–4.01 (m, 12H, CH_2_O), 4.38–4.58 (m, 4H, CH_2_O), 6.25–6.50 (m, 6H, arom. H4, H5, H6); ^13^C NMR δ 24.6 (CH_3_(*t*-Bu)), 28.5 (CH_3_(*t*-Bu)), 29.7 (CH_2_), 37.4 (C(*t*-Bu)), 39.5 (C(*t*-Bu)), 61.4 (CH_2_O), 64.5 (CH_2_O), 98.1 (bisdioxine C1/C5), 102.3 (bisdioxine C4/C8), 104.9 (arom. C4/C6), 117.0 (arom. C5), 146.4 (arom. C1/C3), 163.0 (bisdioxine C3/C7), 169.5 (CO); the aromatic C2 signal was not observed because of broadening owing to the nitrogen quadrupole moment; LC–MS *m*/*z*: 1287.5 [M + H]^+^. The LC–MS indicated the presence of the mono-amine **12** as an impurity, *m*/*z*: 1317.5 [M + H]^+^.

**Tetraoxaadamantane 14:** To a solution of 50 mg of dinitro compound **8** in 1 mL of CH_2_Cl_2_ and 1 mL of glacial acetic acid was added 55 µL of conc. HCl, and the resulting mixture was stirred in a closed flask at rt for 48 h. The CH_2_Cl_2_ was evaporated, and the formed precipitate was purified by DCFC, eluting with CH_2_Cl_2_/MeOH 100:1 to yield 10 mg (20%) of yellow crystals. ^1^H NMR δ 0.68–1.26 (m, 72H, CH_3_(*t*-Bu)), 2.18 (br, 8H, CH_2_), 2.77–2.82 (two signals, 2H, tetraoxaadamantane CH), 4.07–4.45 (m, 16H, CH_2_O), 7.02 (2H, arom. H6), 7.05 (m, 2H, arom. H5), 7.40 (m, 2H, arom. H3); HMBC-2D δ 7.02, 7.05 and 7.40 (arom. H6, H5 and H3), 99.9 (bisdioxine C1/C5), 99.4 (tetraoxaadamantane C1/C3), 100.8 (tetraoxaadamantane C5/C7), 109.7 (arom. C3), 115.5 (arom. C6), 121.8 (arom. C5), 138.8 (arom. C2), 146.6 (arom. C1), 152.0 (arom. C4), 162.5, 162.7 (bisdioxine C3/C7), 167.8 (CO), 168.1 (CO), 174.8 (CO); HMQC-2D δ 24.7, 24.9 (CH_3_(*t*-Bu)), 27.5 (CH_2_), 43.5 (tetraoxaadamantane CH), 60.0 (CH_2_O), 64.0 (CH_2_O), 65.3 (CH_2_O), 109.8 (arom. C3), 115.0 (arom. C6), 121.6 (arom. C5); LC–MS *m*/*z*: 1382.8 [M + H_2_O]^+^; Anal. calcd for C_72_H_104_N_2_O_23_: C, 63.31; H, 7.68; N, 2.05; found: C, 63.65; H, 8.55; N, 1.97.

## Supporting Information

File 1 Drawings of the *R* and *S* enantiomers of **3** and the *R,S (meso)*, *R,R*, and *S,S* diastereoisomers of the bisdioxine macrocyles.

File 2 Assignment of ^1^H NMR spectra and copies of ^13^C NMR spectra of **8**, **11**, and **13**.
