# Supplementary material for: Synthesis of functionalized macrocyclic derivatives of trioxabicyclo[3.3.0]nonadiene
Source: Beilstein J Org Chem. 2012 May 15;8:738–43. doi: 10.3762/bjoc.8.83 (PMC3388861; doi:10.3762/bjoc.8.83)
Supplement: File 1 — Drawings of the R and S enantiomers of 3 and the R,S (meso), R,R, and S,S diastereoisomers of the bisdioxine macrocyles. [file Beilstein_J_Org_Chem-08-738-s001.pdf]

Supporting Information

for

Synthesis of functionalized macrocyclic derivatives of trioxabicyclo[3.3.0]nonadiene

Sabine Leber[1,2], Gert Kollenz*[1] and Curt Wentrup*[2]

Address: [1]Institute of Chemistry, Karl-Franzens University of Graz, Heinrichstrasse 28, A-8010 Graz, Austria and [2]School of Chemistry and Molecular Biosciences, The University of Queensland, Brisbane, QLD 4072, Australia

Email: Gert Kollenz - gert.kollenz@uni-graz.at; Curt Wentrup - wentrup@uq.edu.au

* Corresponding author

Drawings of the *R* and *S* enantiomers of 3 and the *R*,*S* (*meso*), *R*,*R*, and *S*,*S* diastereoisomers of the bisdioxine macrocyles.

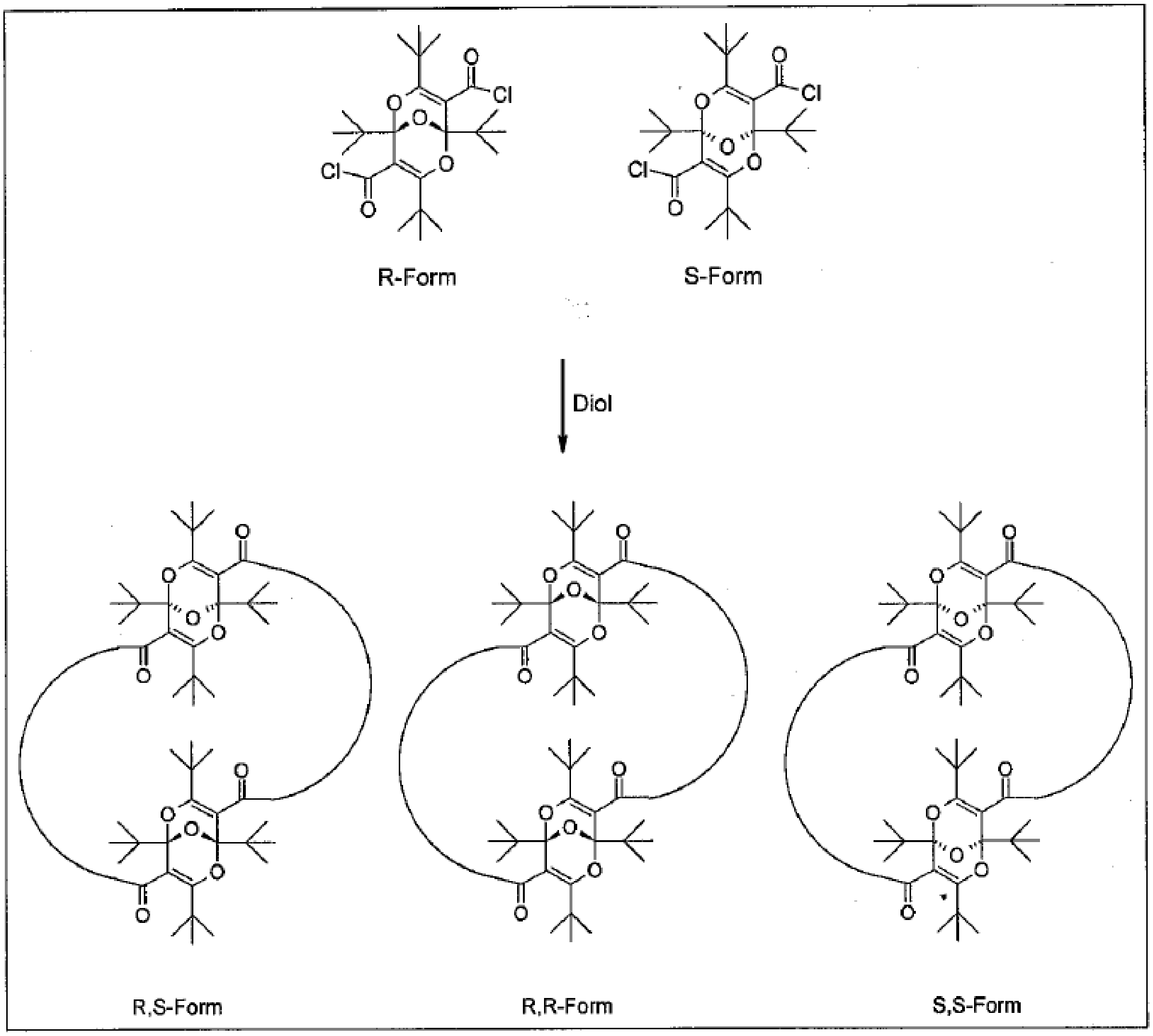

Figure S1: Drawings of the *R* and *S* enantiomers of bisdioxine **3** and the *R*,*S* (*meso*), *R*,*R*, and *S*,*S* diastereoisomers of the bisdioxine macrocyles.
