# Supplementary material for: Synthesis of functionalized macrocyclic derivatives of trioxabicyclo[3.3.0]nonadiene
Source: Beilstein J Org Chem. 2012 May 15;8:738–43. doi: 10.3762/bjoc.8.83 (PMC3388861; doi:10.3762/bjoc.8.83)
Supplement: File 2 — Assignment of 1H NMR spectra and copies of 13C NMR spectra of 8, 11, and 13. [file Beilstein_J_Org_Chem-08-738-s002.pdf]

Supporting Information

for

Synthesis of functionalized macrocyclic derivatives of trioxabicyclo[3.3.0]nonadiene

Sabine Leber[1,2], Gert Kollenz*[1] and Curt Wentrup*[2]

Address: [1]Institute of Chemistry, Karl-Franzens University of Graz, Heinrichstrasse 28, A-8010 Graz, Austria and [2]School of Chemistry and Molecular Biosciences, The University of Queensland, Brisbane, QLD 4072, Australia

Email: Gert Kollenz - gert.kollenz@uni-graz.at; Curt Wentrup - wentrup@uq.edu.au

* Corresponding author

Assignment of ^{1}H NMR spectra and copies of ^{13}C NMR spectra of 8, 11, and 13.

Content:

Figure S2. Assignment of ^{1}H NMR signals for compound **8**........S2

Figure S3. Assignment of ^{1}H NMR signals for compound **11**........S3

Figure S4. ^{13}C NMR spectrum of compound **8**........S4

Figure S5. ^{13}C NMR spectrum of compound **11**........S5

Figure S6. ^{13}C NMR and HSQC $^{1}H/^{13}C$ NMR correlation for compound **13**........S6

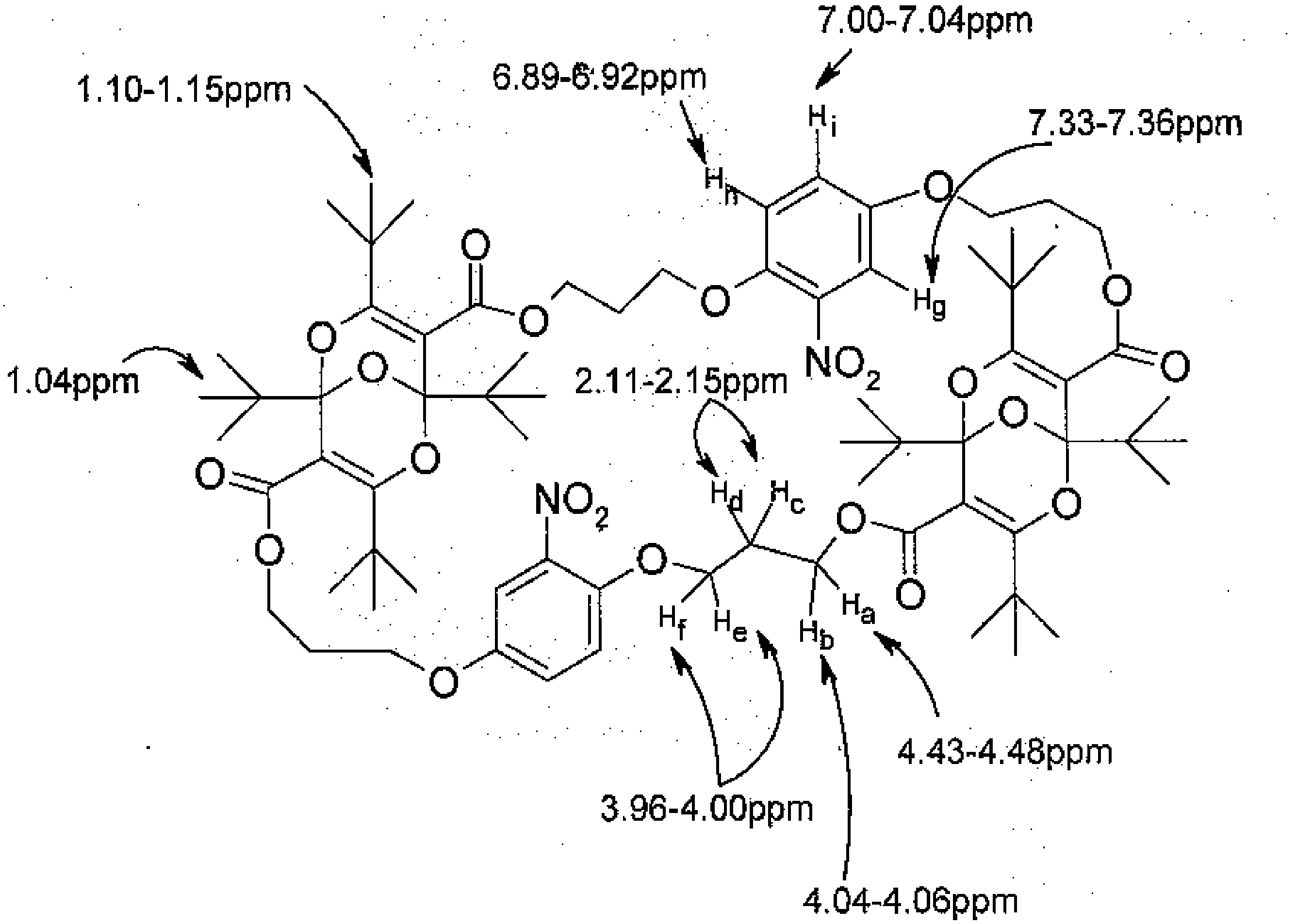

Figure S2: Assignment of ^{1}H NMR signals for compound **8**.

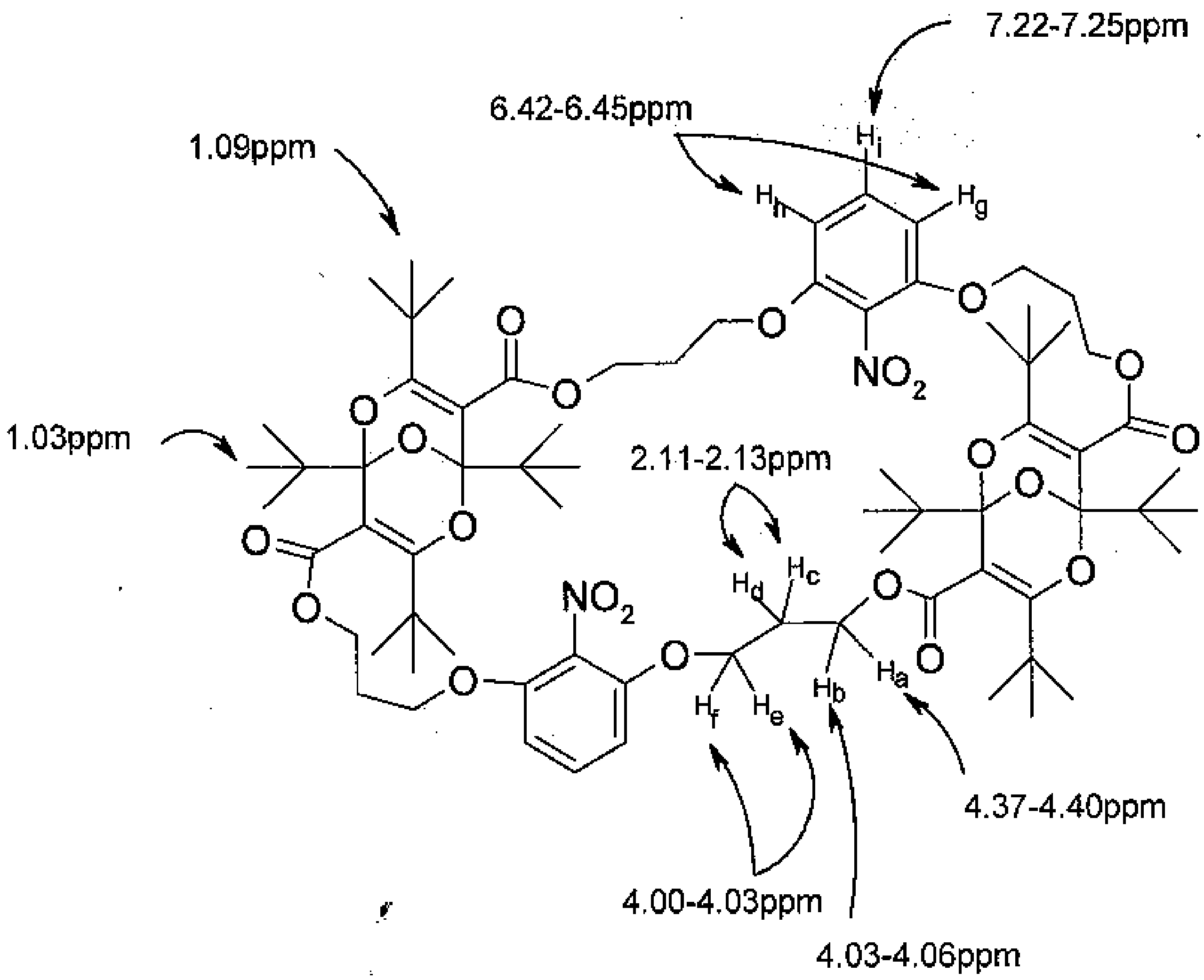

Figure S3: Assignment of ^{1}H NMR signals for compound **11**.

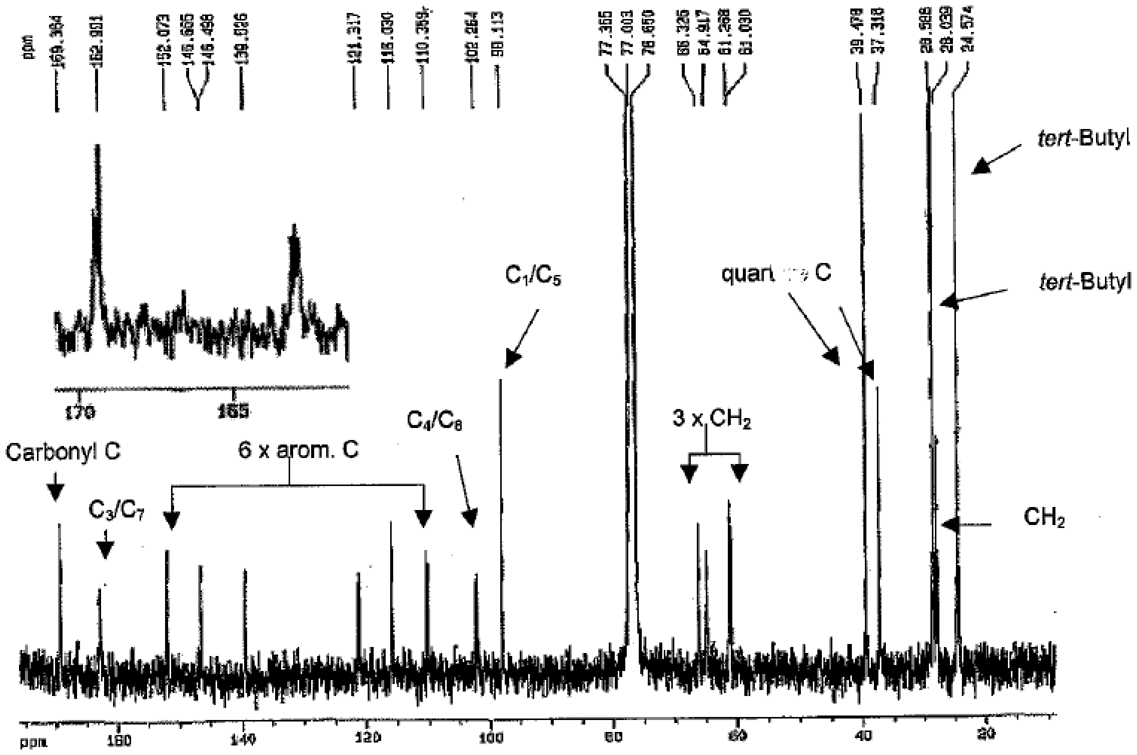

Figure S4: ^{13}C NMR spectrum of compound **8**. C1/C5, C3/C7 and C4/C8 refer to the bisdioxine unit.

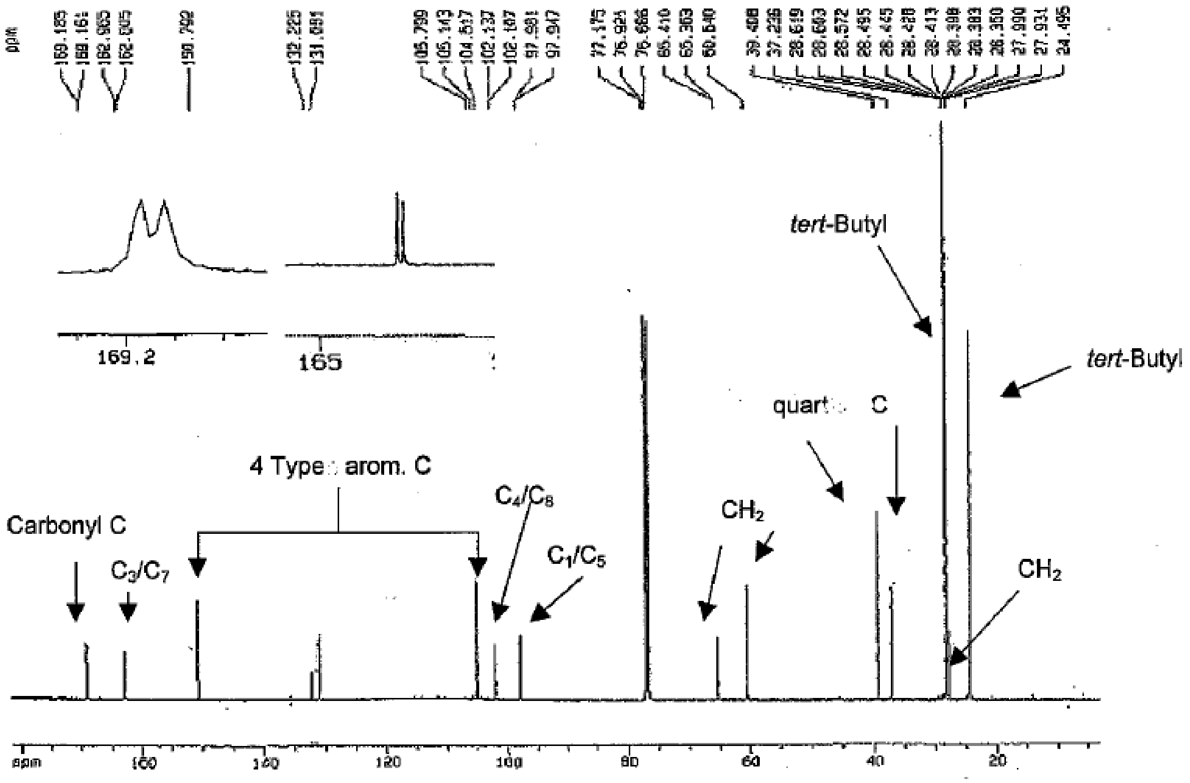

Figure S5: ^{13}C NMR spectrum of compound **11**. C1/C5, C3/C7 and C4/C8 refer to the bisdioxine unit.

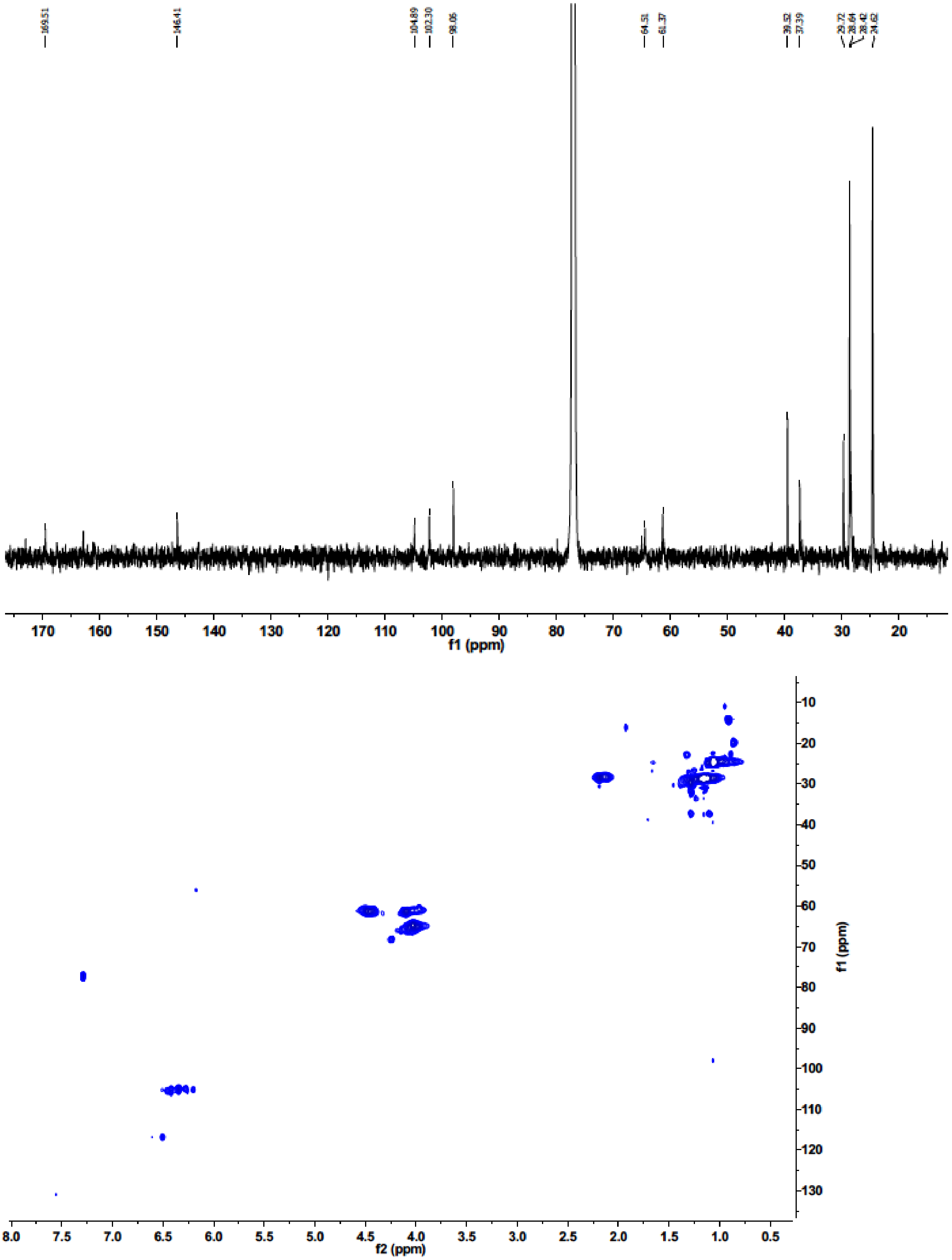

Figure S6: ^{13}C NMR and HSQC ^{1}H/^{13}C NMR correlation for compound **13**.
